# Prenatal Elexacaftor/Tezacaftor/Ivacaftor for Fetal Meconium Ileus: First Italian Case and Narrative Overview of the Emerging Evidence

**DOI:** 10.3390/jcm15072625

**Published:** 2026-03-30

**Authors:** Alessandra Boni, Chiara Vassallo, Fabiana Ciciriello, Luca Cristiani, Alessandro Mancini, Luigi Zucaro, Sonia Graziano, Bianca Maria Goffredo, Federico Alghisi, Massimiliano Raponi, Isabella Fabietti, on behalf of OPBG CF Pregnancy and Fetal Therapy Multidisciplinary Group

**Affiliations:** 1 Pneumology and Cystic Fibrosis Unit, Bambino Gesù Children’s Hospital, IRCCS, 00165 Rome, Italy; fabiana.ciciriello@opbg.net (F.C.); luca.cristiani@opbg.net (L.C.); federico.alghisi@opbg.net (F.A.); 2 Clinical Area of Fetal, Neonatal and Cardiological Sciences and Research Area of Perinatal Medicine—“Bambino Gesù” Children’s Hospital IRCCS, 00165 Rome, Italy; chiara.vassallo@opbg.net (C.V.); isabella.fabietti@opbg.net (I.F.); 3 Division of Metabolic Diseases and Hepatology, Bambino Gesù Children’s Hospital, IRCCS, 00165 Rome, Italy; 4 Bioethical Function, Bambino Gesù Children’s Hospital, IRCCS, 00165 Rome, Italy; luigi.zucaro@opbg.net; 5 Psychology Unit, Child & Adolescent Psychiatry Unit, Bambino Gesù Children’s Hospital, IRCCS, 00165 Rome, Italy; sonia.graziano@opbg.net; 6 Medical Direction, Bambino Gesù Children’s Hospital, IRCCS, 00165 Rome, Italy; massimiliano.raponi@opbg.net; 7 Area of Management, Diagnostic Innovation and Clinical Pathways, Perinatal Surgery Research Unit, Bambino Gesù Children’s Hospital, 00165 Rome, Italy

**Keywords:** meconium ileus, fetal therapy, cystic fibrosis, healthy carrier mother, elexacaftor/tezacaftor/ivacaftor

## Abstract

**Introduction**: Cystic fibrosis (CF) frequently presents prenatally with meconium ileus (MI), a condition associated with significant neonatal morbidity and long-term gastrointestinal complications. The advent of highly effective CFTR modulators, particularly elexacaftor/tezacaftor/ivacaftor (ETI), during pregnancy remains off-label, and their role as in utero therapy for affected fetuses of carrier mothers is still emerging. **Methods**: We conducted a narrative literature review using PubMed, Embase, and Scopus to identify published reports of in utero CFTR modulator therapy for MI between 2022 and 2026. Seven relevant studies were identified and qualitatively synthesized. Their findings were interpreted in comparison with the present case. **Results**: We describe the first Italian case of prenatal ETI therapy for fetal CF. At 32 weeks’ gestation, ultrasound (US) findings were suggestive of evolving MI. Both parents were carriers of F508del CFTR and subsequent testing confirmed fetal homozygosity. Following urgent multidisciplinary consultation and ethics committee approval, maternal ETI therapy was initiated at 33 weeks’ gestation. After 21 days of treatment, follow-up fetal US demonstrated improvement in bowel dilatation and hyperchogenity. The infant was delivered at 36 + 2, passed meconium spontaneously, and required no surgical intervention. Pharmacokinetic assessment showed substantial transplacental transfer of all three ETI components, with cord-to-maternal plasma ratios of 0.34 (elexacaftor), 2.48 (tezacaftor), and 0.58 (ivacaftor), and detectable concentrations in amniotic fluid. Postnatally, sweat chloride was elevated, and pancreatic function transitioned from initially preserved to pancreatic insufficiency within the first month of life. **Conclusions**: This case and literature review suggest that prenatal CFTR modulation may influence the early trajectory of CF, potentially by preventing MI and potentially delaying the progression to pancreatic insufficiency and potentially reducing later gastrointestinal complications. While evidence remains limited, these findings highlight a potential therapeutic window during fetal life and underscore the need for prospective data collection, structured registries, and harmonized clinical guidance in this evolving field.

## 1. Introduction

Cystic fibrosis (CF) is the most common autosomal recessive genetic disorder among Caucasians and is caused by mutations in the CFTR gene [1]. One of the earliest clinical manifestations of CF is meconium ileus (MI), which is a congenital intestinal obstruction caused by thick, sticky meconium in the distal ileum. MI affects 10–20% of neonates with CF and can be potentially detected in the second trimester of pregnancy through intestinal dilatation and hyperechogenicity [2]. The risk is particularly high in pwCF (people with CF) with two copies of the most common F508del mutation, with a 24.9% risk of presenting with MI [3]. Complicated MI occurs in 40–50% of cases and often requires surgical interventions due to severe intrauterine bowel damage, carrying risks of short bowel syndrome and postoperative complications [3]. Moreover, MI is associated with increased long-term gastrointestinal morbidity, including cholestasis related to prolonged parental nutrition and intestinal resection [3,4]. Furthermore, infants who present with meconium peritonitis may experience recurrent intestinal obstruction later in childhood, often attributable to adhesions or segmental volvulus as a consequence of the initial intra-abdominal inflammatory process [3,4]. MI is not merely a neonatal surgical emergency but a marker of long-term intestinal vulnerability in CF. In fact, recurrent intestinal obstruction due to inspissated fecal material, known as distal intestinal obstruction syndrome (DIOS), occurs more frequently in patients with a history of neonatal MI, with a risk of approximately 50% compared with about 15% in the overall pwCF [3].

Although evidence remains limited, available data suggest elexacaftor/tezacaftor/ivacaftor (ETI, known as Kaftrio^®^ in Europe) use during pregnancy appears generally safe for both the mother and the fetus [5]. Ongoing prospective studies, including MAYFLOWERS (NCT04828382), are systematically evaluating maternal and neonatal outcomes associated with prenatal and breastfeeding exposure [5,6]. Despite these advances, the use of ETI as prenatal therapy is still experimental and is not currently recommended by regulatory authorities, underscoring the need for cautious, case-by-case consideration. Nevertheless, recent evidence has described favorable outcomes following prenatal ETI administration to treat severe fetal CF manifestations, such as MI [7].

We present the first Italian case of prenatal ETI administration for fetal meconium ileus, integrating clinical observation with a narrative synthesis of emerging evidence on in utero CFTR modulation, transplacental pharmacokinetics, and the psychological implications of early therapeutic intervention.

## 2. Case Report

### 2.1. Prenatal Presentation and Diagnostic Evaluation

**Figure 1:**
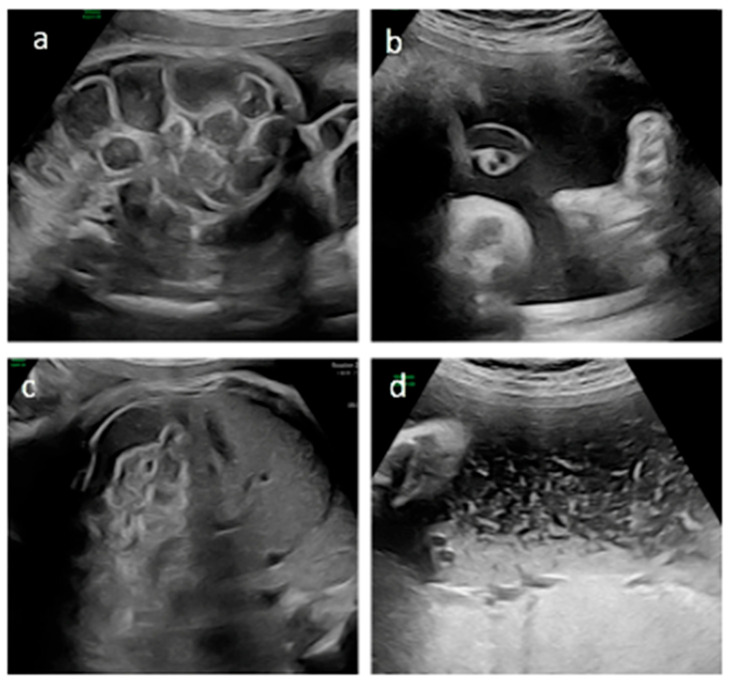
Ultrasound findings of the bowel and amniotic fluid before (**a**,**b**) and after (**c**,**d**) treatment with ETI. At 33 weeks’ gestation, diffuse bowel dilation with increased wall thickness (1.7 mm) and poorly echogenic amniotic fluid are observed (**a**,**b**). At 36 weeks’ gestation, reduced bowel caliber with persistent wall thickening and markedly echogenic amniotic fluid consistent with meconium are observed (**c**,**d**).

We report the case of a 36-year-old primigravida who conceived spontaneously. First-trimester aneuploidy and mid-trimester structural screening were unremarkable. During the third trimester, diffuse dilatation of the fetal bowel loops was detected. Subsequent parental genetic screening revealed that both parents were heterozygous carriers of the F508del mutation in the CFTR gene. The couple declined invasive prenatal testing at that time. The patient was referred to our maternal-fetal medicine unit at 33 + 0 weeks of gestation. Diffuse dilation of the fetal bowel loops was confirmed, with the largest loop measuring 18.2 mm in diameter and a wall thickness of 1.7 mm, with preserved peristalsis (Figure 1).

### 2.2. Prenatal Intervention

A multidisciplinary team (CF specialists, maternal–fetal medicine experts, pediatric surgeons and clinical ethicists) was rapidly convened to evaluate the initiation of maternal ETI therapy despite the absence of regulatory approval for fetal indications [8]. Following extensive discussion, compassionate off-label prenatal treatment was approved, supported by the hospital institution and a local CF patient association and grounded in the ethical principle of “primum non nocere”, given the absence of reported maternal or fetal toxicities with ETI therapy. Before the medication was dispensed, follow-up US demonstrated new onset polyhydramnios, prompting amnioreduction and fetal CFTR analysis. Genetic testing confirmed fetal CF with homozygosity F508del mutation. After informed parental consent and formal ethics committee approval, maternal ETI therapy was started at 33 weeks of gestation using the standard adult dosing regimen. The mother was clinically healthy. The aim of therapy was to improve the fetal condition, specifically to reduce or resolve fetal bowel dilatation due to presumed MI. Treatment was well tolerated, and serial laboratory monitoring showed no clinically significant abnormalities. No maternal adverse effects were observed during the entire treatment period. Between 34 + 0 and 36 + 0 weeks’ gestation, serial US demonstrated progressive stabilization and improvement of both intestinal findings and amniotic fluid volume. At 34 + 0 weeks, the largest bowel loop measured 15 mm with thin walls and preserved peristalsis, amniotic fluid was normal, and fetal growth was appropriate for gestational age. By 36 + 0 weeks, bowel dilatation decreased to 13 mm with increased wall thickness but preserved peristalsis, and amniotic fluid was at the upper limit of normal (Figure 1).

These findings are consistent with a biologically plausible transplacental response to ETI without evidence of maternal toxicity.

### 2.3. Neonatal Outcome

**Figure 2:**
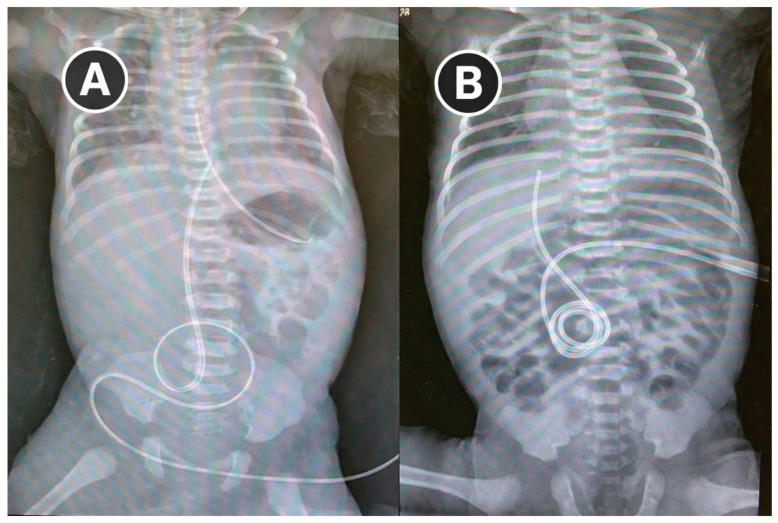
Progressive increase in bowel gas within the intestinal loops in a neonate with uncomplicated meconium ileus. (**A**) Radiograph obtained a few hours after birth, after placement of a nasogastric tube and an umbilical catheter; (**B**) Radiograph obtained 3–4 days later, demonstrating progressive intestinal gas filling.

After 21 days of maternal ETI therapy, vaginal delivery occurred at 36 + 2 weeks without complications. The infant passed meconium shortly after birth, had an uncomplicated transition, and required no surgery. Serial abdominal radiographs over the first four days showed progressive resolution of bowel distension (Figure 2).

Pancreatic enzyme replacement was initially deferred and started only after the first CF outpatient visit. At 10 days, the infant had not regained birth weight; fecal elastase was 204.8 µg/g (pancreatic sufficiency). At 31 days, elastase fell to <50 µg/g, indicating pancreatic insufficiency and sweat chloride at 61 days was 96 mmol/L, confirming CF.

Electrocardiographic and ophthalmologic assessments were normal. Follow-up included routine liver function monitoring and ongoing evaluation of respiratory status, nutrition, growth, and overall clinical course. A longitudinal neurodevelopmental assessment is currently ongoing as a part of the multidisciplinary follow-up.

### 2.4. Pharmacokinetic Assessment

Therapeutic drug monitoring demonstrated maternal systemic exposure: 12 days after ETI initiation, plasma concentrations of elexacaftor (ELX), tezacaftor (TEZ), and ivacaftor (IVA) were 8.59, 0.95, and 0.75 µg/mL. Immediately after birth, cord blood samples were obtained from both the umbilical artery and vein, and a sample of amniotic fluid was collected. ETI concentrations were measured in cord blood plasma and amniotic fluid (Table 1) using a recently published liquid chromatography–tandem mass spectrometry (LC–MS/MS) method [9]. Cord blood concentrations demonstrated substantial transplacental transfer of all three ETI components, with cord-to-maternal ratios (calculated as the ratio between drug concentration in umbilical cord blood and maternal plasma concentration) of 0.34 for ELX, 2.48 for TEZ, and 0.58 for IVA, indicating particularly marked fetal exposure to TEZ.

### 2.5. Psychological Assessment and Follow-Up of the Parental Couple

The parents underwent psychological assessment at 33 weeks’ gestation, with supportive interviews and screening for anxiety and depression according to the International Mental Health Guidelines for Cystic Fibrosis [10]. Anxiety was evaluated using the Generalized Anxiety Disorder-7 (GAD-7) [11] and depression using the Patient Health Questionnaire-9 (PHQ-9) [12], aligned with DSM-5 criteria [13]; both are validated self-report scales scored 0–3 per item, with severity categorized as none (0–4), mild (5–9), moderate (10–14), or severe (≥15). At baseline, both parents reported mild anxiety and depressive symptoms. Postnatal and follow-up assessments showed clinically significant scores. Psychoeducational sessions were provided to support adaptive coping and enhance disease-related knowledge, with evidence of adequate coping strategies.

## 3. Materials and Methods

The aim of this literature search was to summarize the currently available evidence regarding the prenatal use of ETI for the treatment of fetal MI in pregnancies at risk for CF. A targeted search of PubMed/MEDLINE, Embase and Scopus was conducted for studies published between January 2019 and January 2026, reflecting the period during which ETI was widely used by women of reproductive age. The search terms used were combinations of the following: “cystic fibrosis”, “pregnancy”, “CFTR modulators”, “elexacaftor”, “tezacaftor”, “ivacaftor”, “Kaftrio”, “Trikafta”, “prenatal exposure”, and “meconium ileus”. Boolean operators (AND/OR) were used to refine the search and identify studies describing prenatal ETI exposure, especially for MI or related prenatal intestinal manifestations of CF. Studies were eligible if they reported prenatal exposure to ETI during pregnancy and described maternal, fetal or neonatal outcomes. Only English language publications were included. Eligible study designs included case reports, case series, observational studies, registry analyses and pharmacokinetic reports. Excluded studies were if focused exclusively on postnatal ETI exposure and lacked sufficient clinical data on pregnancy and neonatal outcomes.

All identified records (7 papers) were screened by reviewing titles and abstracts. Full-text articles were subsequently assessed for eligibility based on predefined inclusion criteria (7 papers with a total of 31 CF patients with MI in utero). Given the limited number of published reports and the heterogeneity of available literature, findings were synthesized qualitatively rather than quantitatively.

## 4. Results

To our knowledge, this represents one of the earliest successful Italian experiences of prenatal ETI therapy for fetal CF. In our case, early initiation of ETI was associated with stabilization of fetal intestinal abnormalities and may have favorably influenced early disease trajectory, potentially reducing morbidity and hospitalization. This is particularly relevant because MI frequently requires surgical management, with current practice increasingly leaning toward operative intervention rather than conservative approaches [14]. Moreover, MI identifies a subgroup of pwCF predisposed to lifelong gastrointestinal morbidity, including surgical sequelae and DIOS.

The present case provides an opportunity to interpret our clinical observation in the context of the limited evidence currently available regarding prenatal ETI therapy for fetal MI.

The first case of a carrier healthy mother was reported in the literature by Szentpetery et al. in 2022 (USA) [15]. In detail, the authors described the case of a healthy carrier mother (heterozygous F508del) expecting a homozygous F508del female (prenatal invasive diagnosis of CF), who started ETI therapy at 32 weeks of gestation to treat fetal MI detected by US at 23 weeks. After 13 days of treatment, US images still showed residual ileus, but after 27 days of therapy, the obstruction was resolved (disappearance of intestinal dilatation) [15]. Similarly to our case, the CF newborn was born at term and presented at 2 weeks of life with normal fecal elastase values in addition to borderline sweat chloride. However, since the mother continued with ETI assumption during breastfeeding, the authors suggested that a possible ongoing benefit to the newborn could be derived from drug excretion in the breast milk [15]. In contrast to that report, ETI therapy was not continued after delivery in our case because of cost-related constraints, and the infant probably was subsequently found to have a positive sweat test, which may reflect the absence of continued postnatal exposure.

In 2023, Gómez-Montes and colleagues reported the Case of a healthy mother carrying a fetus with MI identified at 24 weeks’ gestation, in whom ETI therapy was initiated at 31 weeks after genetic confirmation of CF by amniocentesis. Eight weeks later, ultrasonography no longer showed evidence of intestinal obstruction [16]. However, in contrast to our case and to that reported by Szentpetery and colleagues, the newborn described by Gómez-Montes et al. exhibited biochemical features of CF, including an elevated immunoreactive trypsinogen level (58.1 ng/mL), a positive sweat chloride test (80 mmol/L), and a low fecal elastase level (58 µg/g), consistent with pancreatic insufficiency [15,16].

These data suggest that both the beginning and duration of ETI fetal treatment can significantly affect the clinical outcomes in newborns. This is consistent with the Report described by Fortner et al, in which a pregnant woman with CF received ETI treatment throughout the whole gestational period. In this Case, the neonate had a false-negative CF result at newborn screening and preserved pancreatic function for a few months [17].

This effect can probably be explained by the action of ETI during the organogenesis phase of pregnancy, as well as by the greater reduction in pancreatic inflammation and by the impact of breast milk feeding. In both our case and a recent case series, the subsequent development of pancreatic insufficiency may suggest a possible withdrawal effect following discontinuation of prenatal ETI exposure [18]. In fact, persistent pancreatic sufficiency was observed in only one patient who continued ETI exposure through breastfeeding, with pancreatic function progressively improving after approximately five months of postnatal exposure via breast milk [18].

However, pancreatic outcomes after prenatal CFTR modulation appear heterogeneous, as pancreatic insufficiency has also been reported despite prolonged prenatal exposure, as described in the case reported by Gómez-Montes and Bonnel et al. [7,15]. Taken together, these observations suggest that prenatal ETI exposure may delay rather than completely prevent the progression toward pancreatic insufficiency, with the final pancreatic phenotype likely influenced by multiple factors, including timing of therapy initiation, duration of fetal exposure, differences in fetal drug exposure, and continuation of CFTR modulation after birth.

A recent Case series by Metcalf et al. (2025) suggested a correlation between the timing of prenatal ETI initiation and fetal outcomes [18]. In particular, the Series describes three different pregnancies in which the fetus with the earliest exposure to ETI presented with complete in utero resolution of MI, whereas the fetus with the shortest exposure period required surgery for severe MI. The second fetus required only minimal postnatal management [18]. Together, these observations support the potential effectiveness of prenatal CFTR modulation and emphasize the importance of promptly initiating treatment once fetal MI is recognized. This reinforces the idea that both timing and duration of exposure are critical factors for fetal intestinal outcomes.

In the PROTECT workshop case series regarding American and European cases, resolution of fetal bowel abnormalities occurred after a mean of approximately 6 weeks of maternal ETI therapy, with responses observed after as little as 11 to 15 days, whereas the poorest outcomes were reported in infants with the shortest or suboptimal treatment exposure [19]. These findings are consistent with the MODUL-CF study, the largest real-world European multicenter cohort, in which ETI initiated at a median of 30 weeks’ gestation, with a median prenatal exposure of five weeks, led to in utero resolution of meconium ileus within approximately two weeks in 6/8 affected fetuses (7). To better contextualize our findings, we summarized previously reported cases of prenatal ETI exposure associated with fetal MI (31 patients), with a success rate of 77% (24/31) (Table 2). A genotype–outcome correlation could not be established based on the available data from the MODUL-CF and PROTECT series. Notably, favorable outcomes have been reported across a range of genotypes, including those traditionally considered more severe, without a clear genotype-specific pattern [7,19].

**Figure 3:**
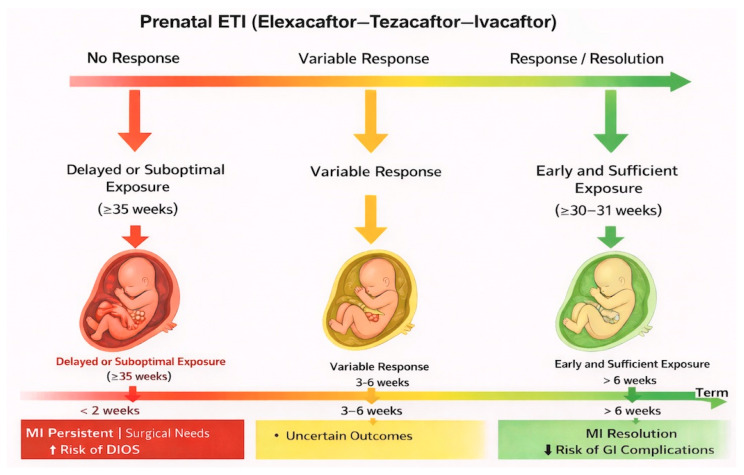
Conceptual model illustrating the impact of timing and duration of prenatal ETI exposure (MI outcomes. Delayed or suboptimal exposure may be associated with persistent MI, whereas early and sufficient exposure (≥30–31 weeks of gestation and >6 weeks of treatment)) is associated with MI resolution and reduced risk of gastrointestinal complications.

As emerging literature suggests, treatment within a window of opportunity at around 30–31 weeks of gestation may be associated with more favorable outcomes without significant adverse effects. The poorest outcomes were observed in infants with the shortest exposure to ETI, which corresponded to a delayed beginning of ETI (35 weeks) [18,19,20] (Figure 3).

As highlighted by the case series reported by Metcalf et al., treatment timing closely correlates with clinical severity. In particular, the third case described in the case series presented additional complications, including jejunal atresia and a meconium pseudocyst, which are recognized markers of complex meconium ileus [18]. In this setting, the combination of delayed initiation of ETI and a more severe underlying intestinal pathology is likely to have contributed to the poor clinical outcome. These observations underscore that, beyond timing of in utero ETI exposure, the intrinsic complexity of MI critically modulates the extent of therapeutic benefit that can be achieved.

Based on the report described by Destoop et al., the reasons behind treatment failures may not be exclusively related to a delay in starting ETI therapy, but also to suboptimal exposure to the drug [20]. Indeed, Destoop and colleagues demonstrated that ETI concentrations measured in both neonatal blood and breast milk were extremely low, raising concerns about variability in placental transfer and excretion in breast milk. Therefore, the authors concluded that a sub-therapeutic dosage of ETI in an overweight mother, as in their case, may have contributed to inadequate fetal exposure, influencing the clinical outcome [20].

Interestingly, a favorable fetal outcome was observed in our case despite the relatively short duration of in utero treatment. This is consistent with the findings reported in the MODUL-CF study and the PROTECT workshop case series [7,19]. Alongside these clinical observations, our pharmacokinetic data extend the findings of Bonnel et al. by revealing a distinct pattern of transplacental ETI transfer, particularly for TEZ. Bonnel et al. first demonstrated the passage of ETI across the placenta, reporting median cord-to-maternal concentration ratios of 0.40 for ELX, 0.54 for IVA and 1.59 for TEZ. This corresponds to approximately 40–50% of maternal levels for ELX and IVA, and 160% for TEZ [7]. In our case, the cord-to-maternal ratios were 0.34 for ELX, 0.58 for IVA and 2.48 for TEZ, which confirms higher fetal exposure to TEZ than to the other two components. Notably, the TEZ ratio observed in our case exceeded that reported by Bonnel et al., although our observation is limited to one mother-infant pair compared with the 13 dyads included in their cohort. Nevertheless, both datasets consistently indicate a preference for the transplacental distribution of TEZ over ELX and IVA. This supports the hypothesis of differential fetal exposure to ETI components and highlights the need for larger pharmacokinetic studies to better define this phenomenon.

In terms of umbilical cord concentrations (Table 1), our data are in line with those predicted and observed in a physiologically based pharmacokinetic model designed to predict maternal and fetal exposures of ETI during pregnancy [21]. This aspect could underlie the reliability of the bioanalytical method used in our Case Report.

The preferential transplacental distribution of TEZ observed may have potential biological significance. TEZ functions as a CFTR corrector, promoting the folding and trafficking of F508del CFTR to the epithelial cell surface. In the fetal intestine, where epithelial CFTR expression is necessary for normal luminal hydration, increased fetal exposure to TEZ could explain the improvement in bowel dilatation and the spontaneous passage of meconium observed. These observations suggest that differential fetal exposure to individual components of ETI during gestation may influence tissue-level responses and their timing, although this hypothesis requires confirmation in larger studies. Moreover, we report direct measurement of all three ETI components in amniotic fluid, an assessment that has not been previously described. To our knowledge, these findings suggest that the amniotic cavity could serve as a pharmacologic reservoir for fetal exposure.

Another potential advantage of fetal therapy in male pwCF involves the development of the vas deferens. Kowalik et al. showed that in a male infant exposed to ETI throughout pregnancy, the vas deferens appeared normal at both 2 weeks and 3 months of age, suggesting that early and sustained fetal exposure to CFTR modulators may be critical for normal duct development [22]. However, findings from Bonnel et al. indicate that exposure to ETI during the third trimester is not alone able to prevent congenital abnormalities of vas deferens. In the MODUL-CF cohort, male infants treated during late gestation still presented with congenital bilateral absence of the vas deferens, confirming that CFTR modulation initiated in the third trimester does not restore vas deferens development, which occurs much earlier in fetal life [7]. Importantly, this therapeutic window is not accessible in carrier mothers, who are asymptomatic and therefore not candidates for CFTR modulator therapy during early pregnancy, further limiting the potential to prevent this developmental defect.

Together, these observations emphasize the potential significance of timing in prenatal CFTR modulation, raising practical and ethical questions about when to initiate treatment in relation to diagnostic certainty. In this context, ETI therapy was initiated before definitive genetic confirmation of CF was reached, following a multidisciplinary, team-based decision-making process and approval by the local Ethics Committee. This was based on the available maternal safety data for ETI and the high pretest probability of CF given the prenatal condition. This approach enabled earlier fetal exposure during a potentially sensitive developmental period, highlighting an evolving ethical framework in which the balance between diagnostic certainty and timely fetal intervention may need to be reconsidered.

Beyond clinical outcomes, the availability of a prenatal therapeutic option can have important ethical and psychological implications. Early access to treatment can transform the experience of parents from passively anticipating disease to actively engaging in decision-making, which could reduce distress during a period of diagnostic uncertainty. This aspect remains underexplored and requires further systematic evaluation.

## 5. Conclusions

The present case, interpreted within the broader context of the available literature, supports the emerging concept that prenatal CFTR modulation may modify the early trajectory of CF in selected high-risk fetuses.

Although the current evidence is limited to case reports and observational studies, converging data, including documented transplacental drug transfer, provide biological plausibility for a direct fetal therapeutic effect.

This emerging approach challenges the traditional model in which CFTR modulators are initiated only after birth, suggesting that timely prenatal intervention may influence early organ-specific disease expression and clinical trajectory.

In the present case, multidisciplinary long-term follow-up is ongoing, including neurodevelopmental assessment, highlighting the importance of systematic monitoring in infants exposed to CFTR modulators during fetal life.

Rather than representing an isolated clinical event, exposure to prenatal ETI may signal the emergence of a new therapeutic window in CF. The development of prospective international registries dedicated to infants exposed to CFTR modulators in utero will be essential to systematically collect long-term safety and developmental data, together with harmonized clinical guidance, before this approach can be considered for broader implementation.

## Figures and Tables

**Table 1:** Concentrations of Elexacaftor, Tezacaftor, and Ivacaftor measured by LC-MS/MS methodology.

Sample Type	Elexacaftor (ELX)	Tezacaftor (TEZ)	Ivacaftor (IVA)
Mother plasma concentration 12 days post ETI initiation (µg/mL)	8.59	0.95	0.75
Plasma concentration from cord blood (Vein) (µg/mL)	3.03	2.33	0.44
Plasma concentration from cord blood (Artery) (µg/mL)	2.88	2.38	0.43
Amniotic Fluid (µg/mL)	0.18	0.74	0.10
Cord-to-maternal plasma concentration ratio	0.34	2.48	0.58

**Table 2:** Published cases of prenatal ETI therapy for fetal meconium ileus.

Author (Year)	Maternal Status	GA at ETI Initiation	Duration of Prenatal Exposure	Genotype of Fetus	Fetal MI Outcome	Pancreatic Status at Birth	Sweat Chloride	Postnatal ETI Exposure
Fortner et al. (2021) [17]	Mother with CF	1 week (continuous)	Entire gestation	F508del/F508del	Complete in utero resolution	Transient PS	False-negative	Continued
Szentpetery et al. (2022) [15]	Healthy carrier	32 weeks	27 days	F508del/F508del	Complete in utero resolution	PS (2 weeks)	Borderline	Continued during breastfeeding
Gómez-Montes et al. (2023) [16]	Healthy carrier	31 weeks	~8 weeks	F508del/F508del	Resolution before birth	PI	Positive	Not specified
MODUL-CF (2025) [7]	Multicenter cohort (8 pt)	Median 30 weeks	Median 5 weeks	F508del/F508del (4 pt) F508del/G542X (1 pt) F508del/G452P (1 pt) F508del/c2959-2960dup (1 pt) F508del/M1V-Q1313k (1 pt)	Resolution (6 pt) No resolution (1 pt) Not evaluated (1 pt)	PI (8 pt)	Not available	Continued during breastfeeding (2 pt)
PROTECT workshop (2025) [19]	Multicenter cases (16 pt)	24 weeks 29 weeks 27 weeks 1 weeks 27 weeks 27 weeks 27 weeks 24 weeks 31 weeks 31 weeks 32 weeks 34 weeks 37 weeks 33 weeks 10 weeks 19 weeks	15 weeks 10 weeks 13 weeks 39 weeks 13 weeks 12 weeks 8 weeks 11 weeks 12 weeks - 6 weeks 5 weeks 1 week 3 weeks 8 weeks 19 weeks	F508del/F508del (10 pt) F508del/N1303K (1 pt) F508del/W1282X (1 pt) F508del/R553X (1 pt) F508del/G542X (1 pt) F508del/5T(12TG) (1 pt) F508del/1811 + 1kbA > G (1 pt)	Resolution (13 pt) No resolution (3 pt) shortest exposure → poorest outcomes	Transient PS (8 pt) PI (6 pt) PS (2 pt)	False negative (1 pt) Borderline (2 pts) Positive (13 pts)	Continued due to neonatal administration (7 pt) Continued during breastfeeding (1 pt)
Metcalf et al. (2025) [18]	Case series (3 pt)	31 weeks 30 weeks 35 weeks	8 weeks 7 weeks < 3 weeks	F508del/F508del (3 pt)	Resolution (1 pt) Resolution after birth (1 pt) No resolution (1 pt) Early/longest exposure → full resolution; shortest → surgery	Transient PS (1 pt) PS (1 pt) PI (1 pt)	Transient false negative (1 pt) Positive (>2 pt)	Continued during breastfeeding (1 pt)
Destoop et al. (2025) [20]	Healthy carrier	27 weeks	11 weeks	F508del/F508del	No resolution	PI	Positive	Discontinued postpartum
Present case (Italy)	Healthy carrier	33 weeks	3 weeks	F508del/F508del	In utero stabilization; no surgery	Transient PS (1 month)	Positive	Discontinued postpartum

Pt = patients, GA = gestational age, PS = pancreatic sufficiency, PI = pancreatic insufficiency.

## Data Availability

The data presented in this study are not publicly available due to privacy and ethical restrictions but are available from the corresponding author on reasonable request.

## Abbreviations

The following abbreviations are used in this manuscript:

CF	Cystic fibrosis
DIOS	Distal ileal obstruction syndrome
ELX	Elexacaftor
ETI	Elexacaftor/tezacaftor/ivacaftor
IVA	Ivacaftor
LC–MS/MS	Liquid chromatography–tandem mass spectrometry
MI	Meconium ileus
pwCF	People with cystic fibrosis
QoL	Quality of life
TEZ	Tezacaftor
US	Ultrasound
